# Dynamic allostery controls the peptide sensitivity of the Ly49C natural killer receptor

**DOI:** 10.1016/j.jbc.2021.100686

**Published:** 2021-04-21

**Authors:** Jiaqi Ma, Cory M. Ayres, Lance M. Hellman, Jason R. Devlin, Brian M. Baker

**Affiliations:** 1 Department of Chemistry & Biochemistry and the Harper Cancer Research Institute, University of Notre Dame, Notre Dame, Indiana, USA; 2 Department of Physical and Life Sciences, Nevada State College, Henderson, Nevada, USA

**Keywords:** allosteric regulation, major histocompatibility complex, protein dynamics, natural killer cells, isothermal titration calorimetry, electrostatics, binding, linkage, thermodynamics, DSF, differential scanning fluorimetry, ITC, isothermal titration calorimetry, MHC, major histocompatibility complex, NK, natural killer, NKD, natural killer domain, OVA, ovalbumin, SPR, surface plasmon resonance, TCR, T cell receptor, VSV, vesicular stomatitis virus

## Abstract

Using a variety of activating and inhibitory receptors, natural killer (NK) cells protect against disease by eliminating cells that have downregulated class I major histocompatibility complex (MHC) proteins, such as in response to cell transformation or viral infection. The inhibitory murine NK receptor Ly49C specifically recognizes the class I MHC protein H-2K^b^. Unusual among NK receptors, Ly49C exhibits a peptide-dependent sensitivity to H-2K^b^ recognition, which has not been explained despite detailed structural studies. To gain further insight into Ly49C peptide sensitivity, we examined Ly49C recognition biochemically and through the lens of dynamic allostery. We found that the peptide sensitivity of Ly49C arises through small differences in H-2K^b^-binding affinity. Although molecular dynamics simulations supported a role for peptide-dependent protein dynamics in producing these differences in binding affinity, calorimetric measurements indicated an enthalpically as opposed to entropically driven process. A quantitative linkage analysis showed that this emerges from peptide-dependent dynamic tuning of electrostatic interactions across the Ly49C–H-2K^b^ interface. We propose a model whereby different peptides alter the flexibility of H-2K^b^, which in turn changes the strength of electrostatic interactions across the protein–protein interface. Our results provide a quantitative assessment of how peptides alter Ly49C-binding affinity, suggest the underlying mechanism, and demonstrate peptide-driven allostery at work in class I MHC proteins. Lastly, our model provides a solution for how dynamic allostery could impact binding of some, but not all, class I MHC partners depending on the structural and chemical composition of the interfaces.

Natural killer (NK) cells are a key component of the immune system’s defense against virally infected and transformed cells. NK cell activity is regulated by signaling through a variety of receptors, the most recognized of which are inhibitory receptors responsible for recognizing class I major histocompatibility complex (MHC) proteins (1). Class I MHC proteins present peptides derived from internally produced proteins to cytotoxic T cells. As long as MHC proteins are appropriately expressed on potential target cells, NK cells remain inhibited. However, class I MHC downregulation can help viruses or tumors escape T cell recognition. The failure of a cell to adequately express class I MHC is thus a disease marker that can be sensed by NK cells, leading to destruction of those cells with reduced capacity to present peptides and be surveilled by cytotoxic T cells.

Class I MHC proteins bind peptides in an extended conformation within their peptide-binding domain, where they are directly recognized by T cell receptors (TCRs) (2). As they exist to detect the presence of the MHC protein rather than specific peptides, NK receptors bind peptide/MHC complexes differently than TCRs, with reduced or, in some cases, no participation of the peptide in the binding interface. For example, the LILR receptors in humans and Ly49 receptors in mice bind class I MHC proteins underneath the peptide-binding domain alongside the MHC α3 domain, forming no direct contacts with presented peptides (3, 4, 5, 6, 7, 8).

Curiously, some NK receptors show sensitivity toward different peptides, despite binding peptide/MHC complexes away from the peptide. One example is the murine Ly49C receptor, which distinguishes between different peptides bound to the class I MHC protein H-2K^b^. In one study, the ovalbumin peptide SIINFEKL (termed OVA) protected H-2K^b^-expressing target cells against Ly49C-mediated NK cell lysis, whereas other peptides, such as the vesicular stomatitis virus peptide RGYVYGQGL (termed VSV), showed reduced protective capacity (9). These findings have been reproduced in other functional studies (10), and biochemical binding experiments confirmed that the binding of Ly49C to H-2K^b^ can indeed vary with the presented peptide (5).

Although the peptide selectivity of Ly49C is well recognized, the underlying mechanism is not understood. As seen with nearly all class I MHC proteins, there are no systematic peptide-dependent structural differences with different peptides bound to H-2K^b^, and the structure of the Ly49C-peptide/H-2K^b^ complex does not reveal any binding-induced conformational changes that might help illuminate a peptide-sensing mechanism (5, 6, 7, 11, 12). Confounding this is the lack of quantitative measurements demonstrating precisely how Ly49C-binding affinity changes with different peptides. In the absence of such data, some investigators have hypothesized the existence of subtle, peptide-dependent conformational or dynamic variances that propagate from the H-2K^b^ peptide-binding groove to the Ly49C-binding site (5, 9, 10).

Recently, we and others have shown that different peptides alter the molecular flexibility of class I MHC proteins (13, 14, 15, 16, 17, 18). The tuning of motional properties directly impacts recognition by TCRs (19), but as motions throughout the protein are impacted, this effect has been also implicated in the binding of other proteins, such as components of the peptide loading machinery (20, 21, 22). Such dynamically driven protein allostery, occurring *via* changes in protein motions that are not apparent in crystallographic structures, is now a well-recognized phenomenon in biochemistry. Examples are found in numerous classes of receptors, enzymes, and signaling modules (23, 24, 25), although the detailed mechanisms are varied and complex, possibly involving phenomena in addition to changes in protein motions, such as concomitant alterations in electrostatics (26). Within the immune system, in addition to class I MHC proteins, dynamic allostery has also been implicated in TCR signaling through its potential to alter TCR association with various signaling domains (27, 28, 29). The lack of a clear structural mechanism that can explain the peptide sensitivity of Ly49C binding to H-2K^b^, together with our prior studies of the motions of MHC proteins, suggests that dynamic allostery could underlie the peptide dependence of Ly49C binding to H-2K^b^.

Here, we studied the peptide dependence of Ly49C. We provide quantitative measurements showing how peptides alter the affinity of Ly49C for H-2K^b^. Our findings revealed subtle changes in *K*_D_ that correlate with peptide protective capacity, demonstrating how the immune system is able to amplify small differences in binding to achieve different biological outcomes (30). Through simulations, we provide evidence that different peptides indeed tune the flexibility of the H-2K^b^ protein. Intriguingly though, calorimetric data indicate the peptide dependence of binding is enthalpically rather than entropically driven. Investigating further, we found that different peptides tune the strengths of Ly49C–H-2K^b^ interfacial electrostatics. These results reinforce previous findings that dynamic allostery can lead to changes in protein electrostatics, with allostery incorporating energetic as well as entropic components (26). Overall, our results provide a quantitative assessment of how different peptides tune Ly49C binding and function, illustrate the underlying mechanism, and demonstrate peptide-driven allostery at work in class I MHC proteins. Lastly, our findings that changes in electrostatics are involved suggest a mechanism for how dynamic allostery can impact some but not all binding partners depending on the structural and chemical makeup of different protein–protein interfaces.

## Results

### Sedimentation velocity verifies specific association between dimeric Ly49C and H-2K^b^

**Figure 1:**
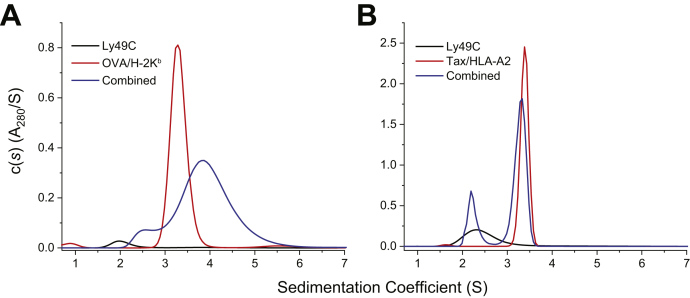
**Sedimentation velocity confirms Ly49C NKD dimerization and its specific binding to H-2K**^**b**^**.***A*, dimeric Ly49C binds OVA/H-2K^b^, as indicated by the shifted peak when the two samples are combined. *B*, Ly49C does not detectably bind the human class I protein HLA-A2, as indicated by the lack of a shifted peak when the two samples are combined.

The Ly49C natural killer domain (NKD) and H-2K^b^ in complex with the ovalbumin peptide (OVA; sequence SIINFEKL) were expressed and purified *in vitro*. We utilized a previously described variant of the Ly49C NKD that differs from wild-type at four residues, which together confer increased stability and higher affinity toward H-2K^b^ while retaining structural identity to the wild-type NKD (5). As Ly49C forms functional homodimers (5, 7), we first assessed protein oligomeric state using sedimentation-velocity analytical ultracentrifugation. Consistent with prior studies (5), we found that at a loading concentration of 4.2 μM, Ly49C NKD alone gave a single peak with a sedimentation coefficient (S value) near 2 and an apparent molar mass of 31 kDa (Fig. 1*A*). As the calculated molecular weight for monomeric Ly49C NKD is 15 kDa, this indicates the presence of a predominantly dimeric species. The OVA/H-2K^b^ complex showed a peak at 3.3 S at a similar loading concentration, yielding an apparent molar mass of 47 kDa, close to its molecular weight of 44 kDa. A mixture of the Ly49C NKD and OVA/H-2K^b^ (4.4 μM and 3.6 μM, respectively) showed a shifted, broad peak with an S value of 4.0, and a shoulder near 2.5 S, consistent with a population distribution that includes a ternary complex and unbound Ly49C and OVA/H-2K^b^. We also performed a control experiment with Ly49C NKD and a human class I peptide/MHC complex (HLA-A2 presenting the Tax_11-19_ peptide) (Fig. 1*B*). This experiment did not yield a shifted peak, confirming the specificity of the Ly49C NKD for murine rather than human class I MHC proteins (31).

### Ly49C sensitivity to peptide is not attributable to different peptide affinities for H-2K^b^

**Figure 2:**
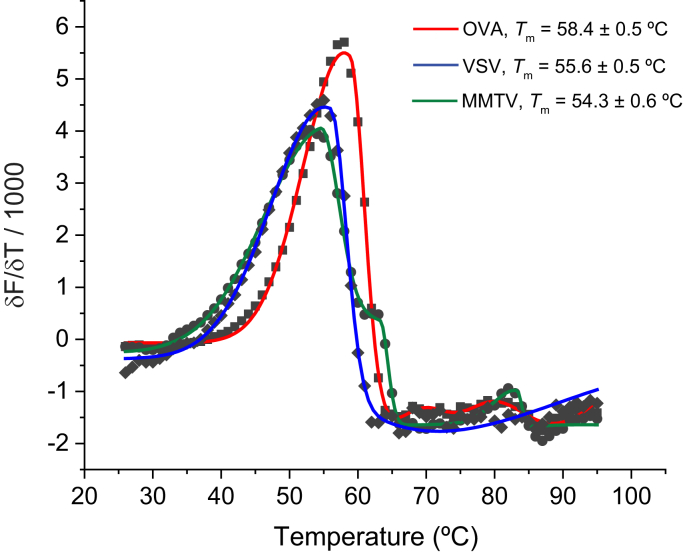
**Differential scanning fluorimetry indicates that the OVA, MMTV, and VSV peptides bind stably to H-2K**^**b**^**.** Data from single representative scans are shown; values represent the average and standard deviations from six independent measurements.

Different peptides show different Ly49C stimulatory capacity in cellular studies. In various experiments, the OVA peptide has been identified as a strong agonist, followed by the MMTV gp70 peptide (MMTV; sequence ANYDFICV), then the vesicular stomatitis virus peptide (VSV; sequence RGYVYQGL) (5, 9, 10). Although variations in peptide-binding affinity to H-2K^b^ have previously been ruled out by studies examining peptide stabilization of H-2K^b^ on cell surfaces (9, 10), we sought a more quantitative assessment to better compare with structural and biophysical data. We thus measured the binding of each peptide to H-2K^b^ using differential scanning fluorimetry (DSF). DSF yields the apparent *T*_m_ of the peptide/MHC complex, which, although describing an irreversible unfolding process, serves as a proxy for the peptide-MHC binding affinity (32, 33). The *T*_m_ values of the three complexes were similar and reflective of high-affinity peptide binding (Fig. 2; *T*_m_ values of 58 °C for OVA, 55 °C for VSV, and 54 °C for MMTV) (32). These results are consistent with the presence of three optimal primary/secondary H-2K^b^ anchor residues for OVA and two for VSV and MMTV (34, 35). For comparison, we previously found that weak binding peptides lacking optimal anchors have *T*_m_ values below 40 °C (32). The small differences in the *T*_m_ values for the OVA, VSV, and MMTV complexes do not track with potency in cellular studies. This data definitively shows that Ly49C peptide sensitivity cannot be attributed to differing strengths of peptide binding to H-2K^b^.

### The affinity of Ly49C NKD for peptide/H-2K^b^ complexes tracks with peptide sensitivity

**Figure 3:**
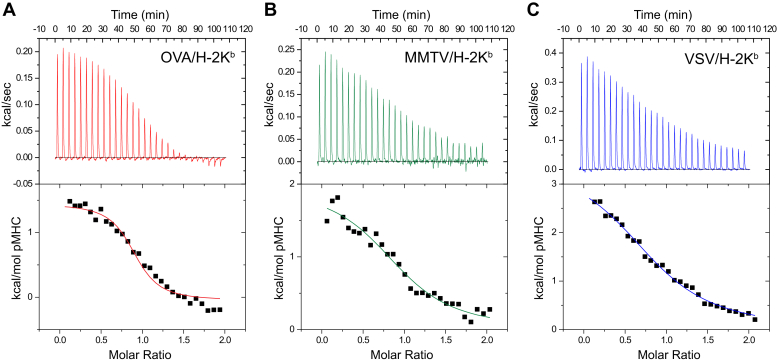
**Isothermal calorimetric titrations of Ly49C NKD with (*A*) OVA, (*B*) MMTV, and (*C*) VSV peptide/H-2K**^**b**^**complexes.** The thermodynamic data, shown in Table 1, indicate subtle differences in binding affinity driven by small changes in thermodynamics.

We next measured the binding affinity (*K*_D_) between the mutant Ly49C NKD and H-2K^b^ presenting the OVA, MMTV, and VSV peptides. Our first attempts utilizing surface plasmon resonance (SPR) indicated a propensity for nonspecific aggregation of Ly49C on SPR sensor surfaces. We thus utilized isothermal titration calorimetry (ITC), which in addition to providing pure solution-based measurements also yields underlying thermodynamic quantities. In the experiments, the Ly49C NKD was in the calorimeter cell at a concentration of 40 μM, ensuring the presence of a stable symmetrical dimer as indicated by analytical ultracentrifugation. Control experiments indicated weak enthalpies at room temperature; titrations were thus performed at 15 °C to improve the signal. The data for each interaction were well fit by a 1:1 binding model with stoichiometries near 1.0, consistent with the independent binding of two peptide/H-2K^b^ complexes to the symmetrical Ly49C dimer (Fig. 3; Table 1). H-2K^b^ presenting the OVA peptide bound the tightest with a *K*_D_ of 1.9 μM, in good agreement with previous work showing *K*_D_ values in the range of 1.7–3.0 μM (5). H-2K^b^ presenting the MMTV peptide bound an intermediate *K*_D_ of 8 μM, and H-2K^b^ presenting the VSV peptide bound the weakest with a *K*_D_ of 12 μM.

**Table 1:** Thermodynamic parameters for Ly49C NKD-binding peptide/H-2K^b^ complexes at 15 °C

Peptide	n	ΔH° (kcal/mol)	ΔS° (cal/K/mol)	ΔG° (cal/mol)	*K*_D_ (μM)
OVA (SIINFEKL)	1.0 ± 0.1	2.0 ± 0.7	33.4 ± 1.7	-7.6 ± 0.2	1.9 ± 0.7
MMTV (ANYDFICV)	1.0 ± 0.2	2.3 ± 0.3	31.4 ± 0.9	-6.7 ± 0.2	8 ± 3
VSV (RGYVYQGL)	1.0 ± 0.1	3.6 ± 0.2	35.1 ± 0.3	-6.5 ± 0.1	12 ± 2

Although the differences in affinity of the three peptide/H-2K^b^ complexes for Ly49C were small, the *K*_D_ values tracked with peptide cellular potency (5, 9, 10). Examining the underlying thermodynamics of the interaction of the Ly49C NKD with the three different peptide/H-2K^b^ complexes showed that the weakening of binding affinity from the OVA to the VSV peptide was driven by changes in both binding enthalpy and entropy, although the changes were small relative to parameter error (Table 1). Focusing on the two peptides with the largest differences in binding affinity as well as inhibitory potency, weaker binding of the Ly49C NKD to the VSV compared with the OVA complex was driven by a shift to a more unfavorable change in ΔH°, with no clear shift in ΔS°.

### Rapid dynamics of the H-2K^b^ protein are altered by different peptides

Previous structural work has failed to identify peptide-dependent structural changes in H-2K^b^ that could explain the peptide sensitivity of Ly49C binding (5, 11, 12). Recently, for the human class I MHC protein HLA-A2, we and others have shown through experiment and computation that peptides can differentially alter the motional properties of the protein. Regions throughout the protein show a peptide dependency, including not only the binding groove but also the α3 domain and the β_2_m subunit (13, 17). Although they have not been studied as extensively as HLA-A2, the motional properties of other class I MHC proteins have shown a similar peptide dependency (18, 36, 37). Given the overall similarities between human HLA-A2 and mouse H-2K^b^, these results suggest the potential for peptide-dependent dynamic allostery in influencing the Ly49C binding.

To assess whether peptide-dependent motional tuning also occurred with H-2K^b^, and if any peptide-associated dynamical changes could be associated with the differences in Ly49C binding, we examined the motions of the H-2K^b^ protein with the OVA and VSV peptides bound using molecular dynamics simulations. Starting with the crystallographic structures of the OVA/H-2K^b^ and VSV/H-2K^b^ complexes (11, 12), we performed 2 μs of molecular dynamics simulations in explicit solvent on each complex. From the simulations, we computed root mean square (RMS) fluctuations of the α carbons (Cα) of each H-2K^b^ residue.

**Figure 4:**
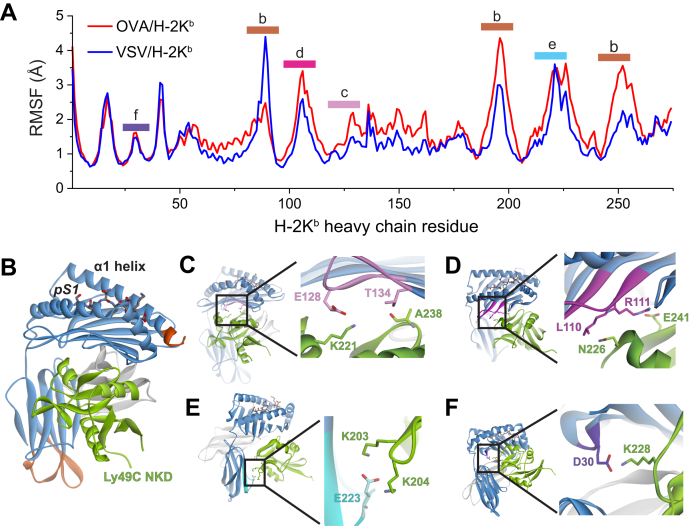
**Molecular dynamics simulations show peptide-dependent variations in H-2K**^**b**^**molecular motions.***A*, Cα RMS fluctuations of the H-2K^b^ heavy chain with the VSV and OVA peptides bound. Peptide-dependent differences are seen across the protein, consistent with prior findings with other class I MHC proteins. Colored bars correspond to the regions highlighted in subsequent panels, which comprise (*B*) the peptide-binding groove and distal loops or (*C–F*) regions that comprise the Ly49C-binding site.

As we saw previously with HLA-A2 (13), Cα atoms throughout the H-2K^b^ protein fluctuated differently depending on whether the OVA or VSV peptide was bound (Fig. 4). Consistent with prior results, the differences were not systematic in that overall protein motions were neither enhanced nor reduced when one or the other peptides was bound. Rather, changes varied across the protein, with some motions enhanced and others dampened depending on peptide, reflecting the complex connections, networks, and overlapping motional pathways frequently seen in allosteric proteins (24, 25, 38). The greatest differences in H-2K^b^ were near the C-terminal end of the α1 helix, as well as loops at the distal end of the protein (Fig. 4*B*). Although these regions lie outside of where Ly49C binds, as found in our prior studies with HLA-A2, more subtle peptide-dependent variations were seen in other regions, including regions that comprise the distributed Ly49C-binding site.

In the structure of Ly49C NKD bound to OVA/H-2K^b^, Ly49C binds to H-2K^b^ underneath the peptide binding groove, on the side of the H-2K^b^ α2 helix (5, 6). This orientation allows Ly49C to interact with residues of H-2K^b^ that form the β sheet floor of the binding groove as well as the α3 domain. Within some parts of this binding site, H-2K^b^ presenting the OVA peptide exhibited slightly higher flexibility. At the base of the H-2K^b^ peptide-binding platform, the stretch of amino acids from Ala125 to Thr132 exhibited RMS fluctuations approximately 0.5 Å greater in the OVA than the VSV simulation. This stretch forms a loop of the H-2K^b^ floor and is a main component of the Ly49C-binding site. For example, Glu128 forms a salt-bridge with Lys221 of Ly49C, and Thr134 hydrogen bonds with Ala238 of Ly49C (Fig. 4*C*). The flexibility of the neighboring H-2K^b^ loop spanning Cys101–Tyr113 was similarly elevated with the OVA peptide. Within this loop, Arg111 forms a salt-bridge with Glu241 of Ly49C, and Leu110 packs against Asn226 of Ly49C (Fig. 4*D*). In the α3 domain, the fluctuations of the "220s loop”, whose dynamics have been shown to be peptide-dependent by both simulation and experiment (13, 18), were similar in the OVA and VSV simulations. In the structure with Ly49C, the 220s loop forms a secondary binding site, with Glu223 forming a salt bridge and hydrogen bond with Lys203 and Lys204 of Ly49C (Fig. 4*E*). Another region of the Ly49C binding site which also behaved similarly in the two simulations was the H-2K^b^ loop containing Glu30, which interacts with Lys228 of Ly49C (Fig. 4*F*). Notably, Glu30 is a key amino acid that influences the specificity of different murine class I MHC proteins for members of the Ly49 family (39). Overall, the molecular dynamics simulations indicate that although the two peptides do differentially tune the dynamics of H-2K^b^, there is no systematic impact on the protein or the Ly49C-binding site, consistent with the observation that the Ly49C-binding entropy change was the same within error regardless of which peptide was bound (Table 1).

### Allosteric modulation of Ly49C binding involves changes in electrostatics

Can dynamic allostery be present without a clear signature in protein motion reflected in simulations or experimentally determined binding entropy changes? Recently, it was shown that dynamic allostery can have a significant energetic (or enthalpic) component resulting from alterations to protein electrostatics (26). In considering this, we were struck by the more favorable ΔH° for Ly49C recognition of the OVA compared with the VSV complex, as well as the participation of several polar or charged amino acids in the Ly49C–H-2K^b^ interface (Table 1 and Fig. 4). We thus hypothesized that peptide-driven allostery could alter the strength of electrostatic interactions across the Ly49C–H-2K^b^ interface. This could result, for example, from altered fluctuations of one or more key charged/polar side chains when the OVA peptide is bound, translating into more optimal electrostatic interactions across the interface.

**Figure 5:**
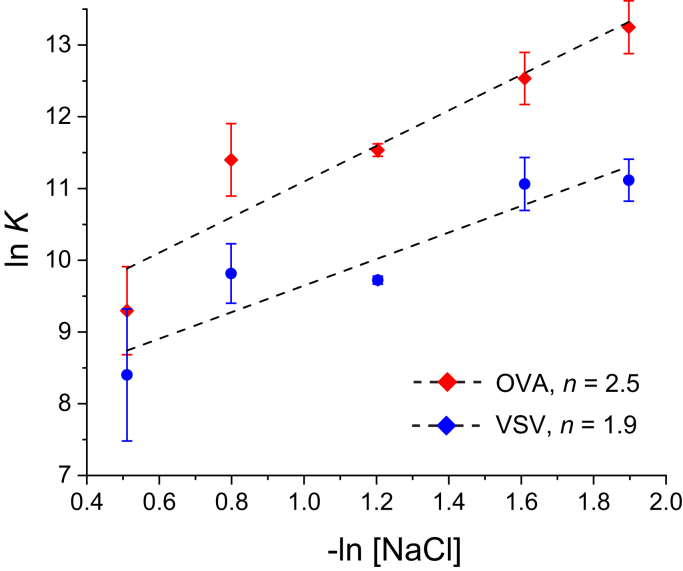
**Linkage analysis of Ly49C NKD binding H-2K**^**b**^**with the OVA and VSV peptides bound.** From thermodynamic linkage, the slopes of the lines give the number of ions released upon binding, which in turn reports on the contribution of electrostatics to binding. The greater slope for the OVA complex indicates stronger electrostatics in the protein–protein interface than in the VSV complex.

To test this hypothesis, we measured the salt dependence of Ly49C NKD binding to the OVA and VSV peptide/H-2K^b^ complexes. The salt dependence of molecular interactions has a long history as an experimental probe of the contributions of electrostatics to binding, with stronger electrostatics revealed by a higher susceptibility to screening through increased ionic strength. We measured affinity as a function of sodium chloride concentration, again using isothermal titration calorimetry to collect data over a range of salt concentrations. This was followed by a thermodynamic linkage analysis of binding affinity versus salt concentration (40). In addition to indicating the effect of increased ionic strength on binding, this analysis reveals the number of ions displaced upon binding, which reports on the role of electrostatics in molecular recognition. The linkage analysis showed that Ly49C NKD binding to the OVA complex indeed involves a stronger electrostatic component, as binding was more effectively screened at higher salt, with a fractionally higher number of ions released (Fig. 5).

## Discussion

Inhibitory NK receptors play a key role in regulating the activity of natural killer cells, which are tasked with eliminating diseased cells in which class I MHC production has been downregulated. Curiously, some NK receptors show a peptide dependence to recognition of class I MHC protein. This behavior is exemplified by the murine protein Ly49C, which recognizes the class I MHC protein H-2K^b^ in a peptide-dependent manner. Although the peptide dependence of Ly49C has been established in functional studies (9, 10), the physical mechanism has remained unclear, particularly as crystallographic structures show Ly49C binding H-2K^b^ “underneath” the peptide-binding groove and forming no direct contacts with the peptide or peptide-flanking MHC α helices (5, 6, 7). Structures of different peptide/H-2K^b^ complexes do not show systematic structural differences that Ly49C may be sensing, and likewise the structures of free and Ly49C-bound H-2K^b^ do not exhibit structural alterations that could give insight into peptide selectivity.

Here, we showed that the functional sensitivity of Ly49C to different peptides is mediated by relatively small changes in receptor-binding affinity: there is only a sixfold difference in *K*_D_ between Ly49C recognition of H-2K^b^ presenting the protective OVA peptide and the less-protective VSV peptide, equivalent to a difference in binding free energy of only 1 kcal/mol. Sensitivity to small changes in binding free energy is a recurring theme in immunology. T cells, for example, are able to distinguish between different ligands despite small variations in TCR affinities, an observation that has led to much discussion about the mechanisms underlying the high sensitivity of immune signaling networks (30). Our data indicate that NK receptors are similarly able to amplify small differences in binding affinity in order to achieve biologically relevant discrimination among different ligands.

It is notable that our measurements were made using a variant of the Ly49C NKD that binds with a stronger affinity. For multiple reasons though, we expect the relative values and the general immunological lessons to be translatable to the wild-type protein. First, the chemical makeup of the Ly49C binding site is identical regardless of the peptide bound to H-2K^b^, so the mutations that distinguish high affinity from wild-type are always placed in the same environment upon binding. Second, the sixfold variation in affinity is consistent with prior measurements: by SPR, Dam *et al.* showed weak binding for wild-type Ly49C recognition of OVA/H-2K^b^, but nearly undetectable for VSV/H-2K^b^ (5). The *K*_D_ for OVA/H-2K^b^ was later reported to be near 100 μM (6). A sixfold reduction to a *K*_D_ near 600 μM would be below the threshold for quantifiable binding by SPR unless the experiment was carefully designed to quantify such a weak affinity (41). Lastly, we recently described an engineered variant of a TCR that binds class I MHC proteins away from the peptide-binding groove (42). Although it binds on the opposing face of the molecule as Ly49C, this engineered TCR shows a peptide dependence similar to what we observe here, providing a benchmark for how peptides can tune the *K*_D_ values for proteins that bind class I MHC proteins away from the peptide-binding groove. We thus conclude that the variation in Ly49C biology with different peptides is indeed achieved with small variations in affinity.

What is the mechanism underlying the changes in Ly49C-binding affinity with peptide? Allostery in the absence of detectable conformational change can proceed *via* ligand-induced changes in coupled molecular motion, which can subtly alter time-averaged structural properties and impact the propensity for conformational variations below the discernment threshold of protein crystallography. These variances in turn can alter the interactions with other binding partners. Such “dynamically driven allostery” is now a well-recognized phenomenon in biochemistry (23, 24, 25), and has been previously implicated in immune recognition and signaling (27, 28, 29). Recently, we and others have shown that different peptides tune the energetic and conformational landscape of class I MHC proteins, impacting motions throughout the protein and highlighting the potential for dynamic allostery (13, 14, 15, 16, 17, 18). Our findings here are thus consistent with a dynamic allostery mechanism: different peptides alter the free energy landscape of H-2K^b^, which in turn impacts the binding affinity of Ly49C. Notably, however, this does not proceed *via* an entropic mechanism as was originally hypothesized for dynamic allostery (43). Rather, it appears that the H-2K^b^ energy landscape is altered such that electrostatic interactions across the interface with Ly49C are strengthened when the OVA peptide is bound. This likely occurs due to subtle changes in the motions of one or more polar or charged groups in interface, such that coulombic or desolvation energetics are shifted in a more favorable direction. This conclusion is consistent with the fact that the interface between Ly49C and H-2K^b^ is comprised of several polar or charged amino acids, as well as recent findings in other systems that, although changes in motion are involved, dynamic allostery can ultimately be enthalpic rather than entropic in nature (26).

A use of energetic components in the mechanism allosteric modulation of Ly49C binding may explain why other proteins that bind class I MHC proteins away from the peptide-binding groove have not shown a clear peptide dependence. Perhaps the best example here is the CD8 coreceptor, whose binding site partially overlaps that of Ly49C, but involves more of the α3 domain (and for CD8αα, the β_2_m subunit) (44, 45). CD8 is generally understood to bind independently of the presented peptide (44, 46). We hypothesize that, compared with Ly49C, CD8 binding relies less on those electrostatic interactions whose motions are modulated by peptide. Generally, differential reliance on electrostatics would provide an elegant solution to how an entire protein’s energy landscape can be tuned by ligand binding, but only select binding partners can be impacted.

How the unique sequences and the physicochemical features of the peptides studied here translate into their specific allosteric effects is currently unknown. In previous work with the human class I MHC protein HLA-A2, we were able to derive predictive models that related specific features such as amino acid size, hydrophobicity, and charge character to dynamic changes (13). This analysis took advantage of a large library of peptide/HLA-A2 crystallographic structures (47). In the absence of such a resource for H-2K^b^, we note that the peptides studied here possess little sequence homology, and the structures of the OVA and VSV complexes display considerable differences in features such as peptide–MHC hydrogen bonding, buried surface, and use of anchor residues (two optimal anchors for VSV; three for OVA). Thus, although exact details regarding how specific peptides tune Ly49C binding to H-2K^b^ awaits further data, based on our work with HLA-A2, the differences observed here are not unexpected.

In conclusion, we have provided quantitative measurements for how different peptides tune the binding of Ly49C to H-2K^b^. The variations in affinity are small, demonstrating the immune system’s ability to amplify relatively small changes in binding free energy to achieve distinct functional outcomes. While the mechanism can be attributed to dynamic allostery, the net effect is energetic in nature, ultimately reflecting alterations in protein electrostatics. Beyond helping connect NK receptor immunology to the biophysics of molecular recognition, our findings provide an example of antigen-dependent allosteric regulation in immune signaling, benchmark the range of affinities that describe class I MHC dynamic allostery, and are relevant for other proteins involved in stimulatory and inhibitory responses in cellular immunity.

## Experimental procedures

### Protein preparation

The Ly49C NKD was expressed in *E. coli* BL21(DE3) cells as inclusion bodies. All experiments utilized a high-affinity mutant that differs from wild-type at four residues (V141K, S171G, E193G, R223K), yielding an increased stability and higher affinity to H-2K^b^ while maintaining the wild-type structure (5). The inclusion bodies were then solubilized in 6 M guanidine and refolded *in vitro* at 4 °C in 400 mM L-arginine with 3.0 mM and 0.8 mM reduced and oxidized glutathione, respectively (5, 29). After incubation overnight, the refolding solution was dialyzed against 10 mM HEPES (pH 7.4) for 48 h at 4 °C. Folded Ly49C NKD was purified by cation-exchange followed by size-exclusion chromatography. The H-2K^b^ heavy chain and murine β_2_-microglobulin were also expressed in *E. coli* as inclusion bodies and refolded at a 1:3 ratio in the presence of 30 mM OVA, VSV, or MMTV peptide at 4 °C, respectively. Refolding buffer for the peptide/H-2K^b^ complexes was 400 mM L-arginine, 100 mM Tris-HCl (pH 8.3), 2 mM EDTA, and 0.2 mM PMSF with 6.3 mM cysteamine and 3.7 mM cystamine. After dialysis against 10 mM Tris-HCl for 48 h at room temperature, the folded peptide/H-2K^b^ complexes were purified by anion exchange followed by size-exclusion chromatography. HLA-A2, used as a control in analytical centrifugation, was produced similarly, using human β_2_-microglobulin and the Tax_11-19_ peptide (sequence LLFGYPVYV). Peptides were purchased commercially from Genscript or AAPTEC.

### Analytical ultracentrifugation

Sedimentation-velocity analytical ultracentrifugation was performed on a Beckman Coulter XL-I instrument at 20 °C using absorbance optics. Rotor speed was 40,000 rpm. Sample sectors were loaded with 360 μl of 4.2 μM Ly49C NKD, 3.7 μM OVA/H-2K^b^, and a mixture of the two. A control experiment was performed using the Tax_11-19_/HLA-A2 complex in place of the H-2K^b^ complex at the same concentration. All samples were prepared in 10 mM HEPES (pH 7.4), 150 mM NaCl, 3 mM EDTA, and 0.005% surfactant P20. Data was analyzed by fitting results to the Lamm equation implemented in SEDFIT (48). Buffer densities and viscosities were calculated using SEDNTERP (49).

### Differential scanning fluorimetry

Thermal denaturation of the peptide/H-2K^b^ complexes was performed using differential scanning fluorimetry as previously described, using a StepOnePlus RT-PCR instrument (32). A 96-well plate was loaded with mixtures of 2 μl 100X SYPRO Orange and 18 μl peptide/H-2K^b^ complex at 15 μM in 10 mM HEPES, 150 mM NaCl, 3 mM EDTA, 0.005% surfactant P20, pH 7.4. The excitation and emission wavelengths for the measurement were set at 587 nm and 607 nm, respectively. Temperature was scanned from 20 °C to 95 °C at a constant rate of 1 °C/min. Data were analyzed in OriginPro 2019. Melting temperatures were determined by fitting the temperature derivative of each melting curve to a bi-Gaussian function. Reported values are the averages and standard deviations of six independent measurements for each complex.

### Isothermal titration calorimetry

Isothermal titration calorimetry was performed at 15 °C using a Microcal VP-ITC. The sample cell was loaded with 1.4 ml Ly49C NKD at an initial concentration of 40 μM. The injection syringe was filled with 300 μl peptide/H-2K^b^ complex at a concentration of 360 μM. Protein in the cell and syringe were equilibrated in 10 mM HEPES, pH 7.4, with NaCl concentrations ranging from 150 mM to 600 mM. Background titrations were performed by titrating the peptide/H-2K^b^ complex into buffer. The volume for each injection was 10 μl, and the injections were performed over 20 s with an interval time of 220 s. Data were processed with the Origin software distributed with the instrument. Data were fit to a 1:1 binding model. To increase accuracy, replicated experiments using the same stock of Ly49C were fit globally, with a shared stoichiometry and local values for ΔH° and *K*_D_. Each titration was repeated three times; equilibrium constants and thermodynamic parameters are reported as average ± one standard deviation of the three measurements. Thermodynamic linkage between Ly49C binding and ion release was performed by linear regression of the natural logarithm of the binding equilibrium constant (1/*K*_D_) versus the negative natural logarithm of the salt concentration, with the slope of the line indicating the number of ions released upon binding (40).

### Molecular dynamics simulations and analysis

Molecular dynamics simulations were performed as previously described (47). Briefly, simulations used Amber18 with GPU acceleration (50, 51). The structures for OVA/H-2K^b^ (PDB code 1VAC) and VSV/H-2K^b^ (PDB code 2VAA) were utilized as the starting coordinates for each simulation (11, 12). Simulations used the ff14SB force field (52). Peptides were modeled with charged terminal residues and sodium ions were added as the counterions to neutralize the system. Complexes were solvated in an isometric box of SPC/E water. After initial energy minimization, both systems were heated to 300K through Langevin dynamics. Solute restraints were gradually relaxed under constant pressure from 25 to 0 kcal mol^-1^ Å^-1^. 50 ps of NVT simulation was then performed, followed by production trajectories. Production trajectories were calculated under constant volume with a 2 fs time step for 2 μs.

## Data Availability

All data are presented in the article and available in raw form from the corresponding author upon reasonable request.

## Conflict of interest

The authors declare that they have no conflicts of interest with the contents of this article.
